# Development and validation of a nomogram for the prediction of brain metastases in small cell lung cancer

**DOI:** 10.1111/crj.13615

**Published:** 2023-04-18

**Authors:** Weiwei Li, Can Ding, Wei Sheng, Qiang Wan, Zhengguo Cui, Guiye Qi, Yi Liu

**Affiliations:** ^1^ Department of Pulmonary and Critical Care Medicine, Shandong Provincial Hospital Shandong University Jinan Shandong 250021 China; ^2^ Shandong Key Laboratory of Infections Respiratory Disease, Medical Science and Technology Innovation Center Shandong First Medical University & Shandong Academy of Medical Sciences Jinan Shandong 250117 China; ^3^ Department of Critical Care Medicine The 960th Hospital of the PLA (People's Liberation Army) Joint Logistics Support Force Jinan Shandong 250012 China; ^4^ Department of Pulmonary and Critical Care Medicine Central Hospital Affiliated to Shandong First Medical University Jinan Shandong 250013 China; ^5^ Cancer Centre Shandong Provincial Hospital Affiliated to Shandong First Medical University Jinan Shandong 250021 China; ^6^ Center of Cell Metabolism and Disease, Jinan Central Hospital Shandong University Jinan Shandong 250013 China; ^7^ Department of Environmental Health University of Fukui School of Medical Science Fukui Japan; ^8^ Department of Medical Engineering Management Shandong Provincial Hospital Affiliated to Shandong First Medical University Jinan Shandong 250021 China; ^9^ Department of Allergy, Department of Pulmonary and Critical Care Medicine Shandong Provincial Hospital Affiliated to Shandong First Medical University Jinan Shandong 250021 China

**Keywords:** brain metastases, nomogram, prophylactic cranial irradiation (PCI), retinol‐binding protein (RBP), small cell lung cancer

## Abstract

**Introduction:**

The aim was to develop and validate a nomogram for the prediction of brain metastases (BM) in small cell lung cancer (SCLC), to explore the risk factors and assist clinical decision‐making.

**Methods:**

We reviewed the clinical data of SCLC patients between 2015 and 2021. Patients between 2015 and 2019 were included to develop, whereas patients between 2020 and 2021 were used for external validation. Clinical indices were analysed by using the least absolute shrinkage and selection operator (LASSO) logistic regression analyses. The final nomogram was constructed and validated by bootstrap resampling.

**Results:**

A total of 631 SCLC patients between 2015 and 2019 were included to construct model. Gender, T stage, N stage, Eastern Cooperative Oncology Group (ECOG), haemoglobin (HGB), the absolute value of lymphocyte (LYMPH #), platelet (PLT), retinol‐binding protein (RBP), carcinoembryonic antigen (CEA) and neuron‐specific enolase (NSE) were identified as risk factors and included into the model. The C‐indices were 0.830 and 0.788 in the internal validation by 1000 bootstrap resamples. The calibration plot revealed excellent agreement between the predicted and the actual probability. Decision curve analysis (DCA) showed better net benefits with a wider range of threshold probability (net clinical benefit was 1%–58%). The model was further externally validated in patients between 2020 and 2021 with a C‐index of 0.818.

**Conclusions:**

We developed and validated a nomogram to predict the risk of BM in SCLC patients, which could help clinicians to rationally schedule follow‐ups and promptly implement interventions.

## Graphical abstract

In this study, we constructed a nomogram to predict the probability of brain metastasis in SCLC patients. We eventually identified 10 factors, including gender, T stage, N stage, ECOG, HGB, LYMPH, PLT, RBP, CEA and NSE, which are readily available in clinical practice.

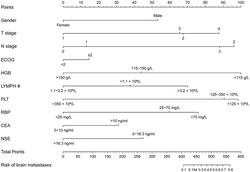

**Abbreviations**
#: absolute value
A/G: ALB/GLO
AAPR: albumin‐to‐alkaline phosphatase ratio
ADA: adenosine deaminase
ALB: albumin
ALP: alkaline phosphatase
ALT: alanine aminotransferase
AST: aspartate aminotransferase
AUC: area under the receiver operating characteristic curve
BM: brain metastases
BMG: β_2_‐microglobulin
C1q: complement C1q
CA125: carbohydrate antigen 125
CEA: carcinoembryonic antigen
CI: confidence interval
CO_2_
: nitrogen dioxide
CRP: C‐reactive protein
CYFRA211: non‐small cell lung cancer associated antigen
DCA: decision curve analysis
ECOG: Eastern Cooperative Oncology Group
GLDH: glutamate dehydrogenase
GLO: globulin
GLU: glucose
HGB: haemoglobin
IQR: interquartile range
LASSO: the least absolute shrinkage and selection operator
LMR: lymphocyte‐to‐monocyte ratio
LYMPH: lymphocyte
mGPS: inflammation‐based prognostic scores
MONO: monocyte
N: node
NA: not applicable
NEUT: neutrophils
NLR: neutrophil‐to‐lymphocyte ratio
NSCLC: non‐small cell lung cancer
NSE: neuron‐specific enolase
OR: odds ratio
OS: overall survival
PCI: prophylactic cranial irradiation
PD‐L1: programmed cell death‐Ligand 1
PFS: progression‐free survival
PLR: platelet‐to‐lymphocyte ratio
PLT: platelet
RBC: red blood cell
RBP: retinol‐binding protein
SA: sialic acid
SCLC: small cell lung cancer
SII: systemic immune inflammation index
SOD: superoxide dismutase
T: tumour
WBC: white blood cell
β: beta coefficient

## 1 INTRODUCTION

Small cell lung cancer (SCLC) is a kind of neuroendocrine tumour with high proliferation rate and enhanced invasiveness, accounting for 13%–15% of all lung cancers.^1^, ^2^ There were approximately 250 000 newly diagnosed SCLC cases, of which patients with brain metastases (BM) account for 15%–20% at initial diagnosis, and mortality from SCLC at least 200 000 each year.^1^, ^3^, ^4^ A recent study suggested that the health care burden is soaring, which was related to lacking of early prevention and treatment in SCLC patients with BM.^5^

The blood–brain barrier creates a natural sanctuary for tumour cells, which blocked drug access to the brain, patients with SCLC are prone to suffer from BM.^6^ Prophylactic cranial irradiation (PCI) is recommended to SCLC patients to prevent and treat BM.^7^ However, PCI is not suitable for all SCLC patients to prevent BM, due to the presence of overtreatment and some adverse events, including anorexia, nausea, impaired quality of life and significant cognitive impairment.^3^, ^7^, ^8^

A clinical prediction model could evaluate the risk of disease, and the benefit of treatment has become the cornerstone of modern clinical practice.^9^ Compared with traditional independent risk factor to assess the metastasis in cancer patients, nomograms have a higher accuracy to predict and diagnose the metastasis in cancer patients. To sum up, predicting the risk of early BM is necessary because it could assist clinician to make better decisions to prevent the risk of BM. Recent evidences have found several predictors that were involved in BM development in SCLC patients, but their specificity and sensitivity were unsatisfactory. Therefore, our aim was to develop a more intuitive, objective and accurate predictive model to identify SCLC patients with high risk of BM.

## 2 METHODS

### 2.1 Source of data

**FIGURE 1:**
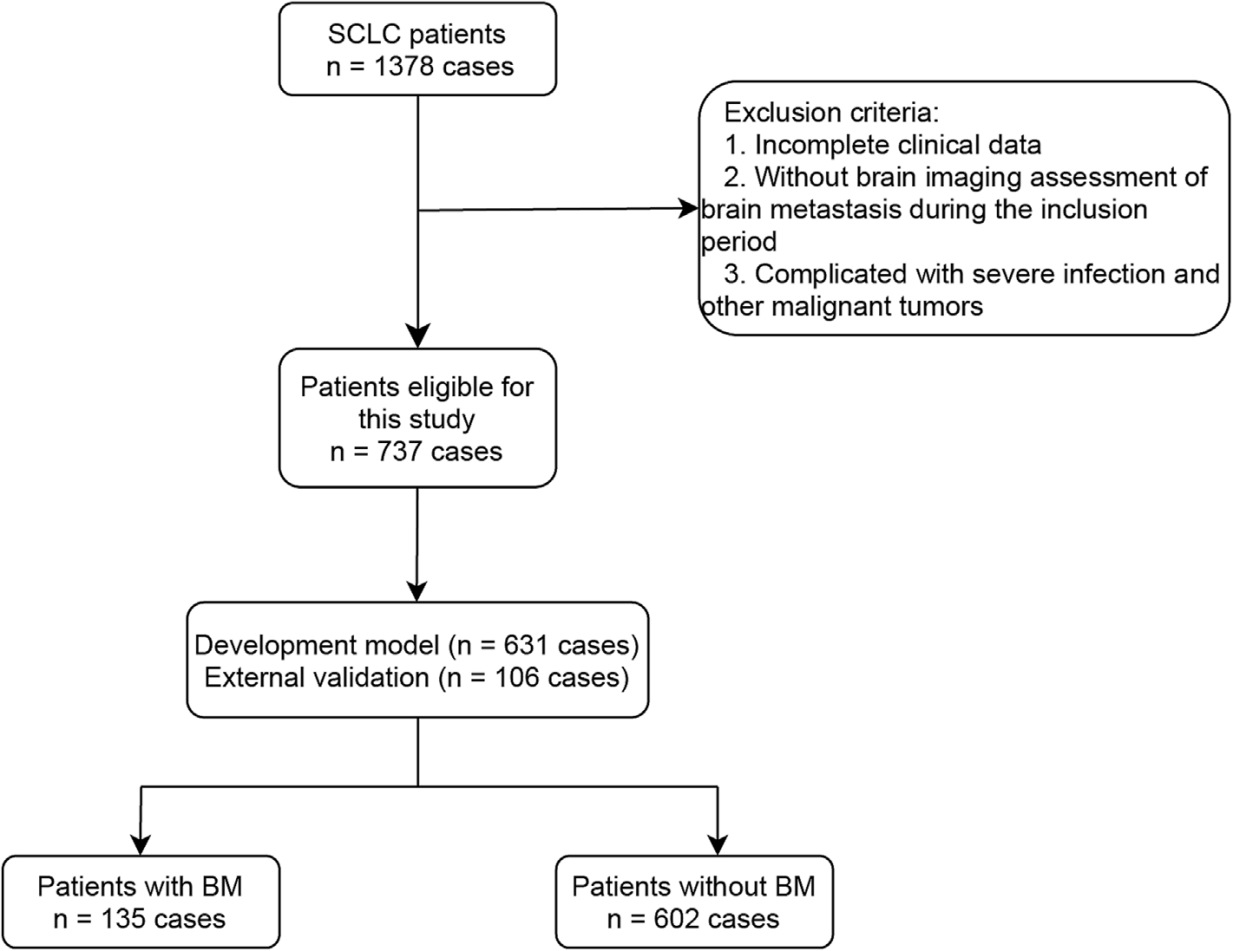
Flowchart of small cell lung cancer (SCLC) patient selection.

We retrospectively reviewed patients who visited the Shandong Provincial Hospital from January 2015 to December 2021 via the electronic medical record system. The inclusion criteria were as follows: (1) SCLC was the primary tumour, which was confirmed by histological or cytological evidence; (2) There was a continuous record of diagnosis and treatment; (3) Imaging data such as computed tomography (CT), magnetic resonance imaging (MRI) or positron emission tomography‐CT (PET‐CT) were used to confirm the occurrence of BM. We excluded patients with incomplete clinical data (the 8th edition TNM stage,^10^ blood routine results, carcinoembryonic antigen, retinol binding protein, etc.); patients with concurrent serious infections or other cancers were also excluded. And we excluded cases with BM without imaging evidence. Finally, there were 737 SCLC patients who met the inclusion criteria and were enrolled in the study from all 1378 SCLC patients (Figure 1), 135 (18.3%) of whom presented with BM. The training cohort of 631 patients between 2015 and 2019 were included to construct the model, and the validation cohort of 106 patients between 2020 and 2021 were used for external validation. This study was approved by the Shandong Provincial Hospital Medical Ethics Committee (Ethical Review of Medical Research on Human Being No. 2020‐301).

### 2.2 Definitions and assessment of variables

We collected the patients' demographic characteristics (age, gender, weight changes and smoking history), serological indices (blood routine, D‐dimer, C‐reactive protein [CRP], liver and renal functions, blood biochemistry and tumour markers), clinicopathological characteristics (TNM stage, primary lesions, Eastern Cooperative Oncology Group [ECOG],^11^ modified Glasgow prognostic score [mGPS]^12^, ^13^) and specific indices (neutrophil‐to‐lymphocyte ratio [NLR], systemic immune‐inflammation index [SII], platelet‐lymphocyte ratio (PLR), lymphocyte‐to‐monocyte ratio [LMR] and albumin‐to‐alkaline phosphatase ratio [AAPR]). Except for NLR, SII, PLR, LMR and AAPR, all other indicators were transformed into categorical variables in this study.

Blood routine included the counts of red blood cells (RBCs), haemoglobin (HGB), platelet (PLT), as well as white blood cells (WBCs) including neutrophils (NEUT), lymphocytes (LYMPHs) and monocytes (MONOs). Liver function was indicated by the levels of aspartate aminotransferase (AST), alanine aminotransferase (ALT), alkaline phosphatase (ALP), adenosine deaminase (ADA), sialic acid (SA), glutamate dehydrogenase (GLDH), albumin (ALB), globulin (GLO), ALB/GLO (A/G) and superoxide dismutase (SOD). Furthermore, renal function indices included β2‐microglobulin (BMG), retinol‐binding protein (RBP) and complement C1q (C1q). Blood biochemistry indices included glucose (GLU), carbon dioxide (CO_2_) and sodium concentration (Na^+^). Finally, tumour markers included carcinoembryonic antigen (CEA), non‐small cell lung cancer associated antigen (CYFRA211) and neuron‐specific enolase (NSE).

The model employed a dichotomous categorical response variable for BM status, dichotomized into with BM and without BM. Weight changes were recorded regardless of whether patients have lost weight or not. TNM stage was determined according to the American Joint Committee on Cancer TNM staging system (8th edition). ECOG was divided into <2 and ≥2 to assess performance status. mGPS was calculated using the levels of serum albumin and CRP to assess systemic inflammation. For CRP >10 mg/L, 1 point was given if ALB value was normal, and 2 was given when ALB <35 g/L. Whereas, 0 point was awarded regardless of ALB values, as long as CRP was normal. In the study BM population, all indices were extracted from the records of first diagnosis. In the study population with BM, all indices were extracted from the records within 24 h of admission of the first diagnosis. In addition, SCLC‐related factors within 24 h after admission in patients without BM were collected from the first diagnostic records of SCLC.

### 2.3 Statistical methods and analyses

Baseline characteristics of the enrolled population were presented as median (interquartile range, IQR) for continuous variables, or as numbers and percentages for categorical variables. The Wilcoxon–Mann–Whitney test was used to compare statistical differences of non‐normally distributed variables between the SCLC patients with and without BM. Categorical variables were analysed using chi‐square test. A two‐sided *p*‐value of <0.05 was considered as the significance threshold for all statistical analyses.

The selection of significant variables relied on the results of univariate logistics regression analysis, clinical importance and predictors identified in previously published articles. We then extracted the following risk factors for the prediction model: gender, HGB, the absolute value of lymphocyte (LYMPH #), PLT, RBP, CEA, NSE, tumour (T) stage, node (N) stage and ECOG. Next, we used the least absolute shrinkage and selection operator (LASSO) method to select the optimal variables with non‐zero coefficients as potential predictors and avoid overfitting of this model. Above factors were selected to develop the final nomogram. β (the regression coefficient), odds ratios (ORs) with 95% confidence intervals (CIs) and *p*‐value were calculated and recorded. The performance of the nomogram was assessed by discrimination, calibration and clinical usefulness in succession. The predictive discriminative ability of the model was displayed by the C‐index and was equivalent to the area under the receiver operating characteristic curve (AUC). Similar to the AUC, the C‐index ranging from 0.5 (no relationship) to 1.0 (perfect concordance) was also used. The calibration plot and Hosmer–Lemeshow test were applied for evaluating calibration. The decision curve analysis (DCA) was displayed to determine the clinic usefulness of the model by quantifying the net benefit at disparate threshold probabilities. The training cohort underwent 1000 bootstrap resamples for internal validation, and external validation was performed on the nomogram by the validation cohort. Finally, we showed the predictive risk points of each predictive risk factor in the nomogram. In addition, the predictive potentials of different cut‐off values for BM in SCLC patients' probability in the nomogram were evaluated by calculating the sensitivity and specificity. The study adhered to the TRIPOD (Transparent Reporting of a multivariable prediction model for Individual Prognosis Or Diagnosis) statement for reporting^14^ and completed the checklist (Data S1).

All statistical analyses were conducted using SPSS, version 26 (SPSS Inc.; Chicago, Illinois) and R language, version 4.0.3 (The R Foundation for Statistical Computing, Vienna, Austria; http://www.r-project.org/).

## 3 RESULTS

### 3.1 Patient characteristics

A total of 737 SCLC patients were included in our study (Figure 1), and BM were confirmed in 135 (18.3%) of them. In the training cohort patients was ranging in age from 25 to 80 years. And most patients (73.4%) were male. No significant differences were found in demographic characteristics (age, smoking history and weight changes) and haematological indices (the values of WBC, neutrophils, monocytes, D‐dimer, AST, ALT, ALP, ADA, SA, GLDH, ALB, GLO, A/G, SOD, CRP, BMG, C1q, Na^+^, CO_2_, CA125, CYFRA211, PLR, NLR, LMR, SII, AAPR, mGPS and the percentages of neutrophils and monocytes) between the two groups (*p* > 0.05). The partly baseline characteristics of SCLC patients with BM or without BM subgroups are summarized in Table 1 (the integrated can be seen in Table S1).

**TABLE 1:** Baseline features of SCLC patients with BM and without BM subgroups.

Parameters	Total (*N* = 631)	With BM (*N* = 103)	Without BM (*N* = 528)	*p‐*value
Age, (years)				0.239
Median [IQR]	61 [54–66]	60 [53–65]	61 [54–67]	
WBC, (×10^9^/L)				0.210
<3.5	42 (6.7%)	10 (9.7%)	32 (6.1%)	
3.5–9.5	532 (84.3%)	87 (84.5%)	445 (84.3%)	
>9.5	57 (9.0%)	6 (5.8%)	51 (9.7%)	
RBC, (×10^12^/L)				<0.001
<3.8	102 (16.2%)	31 (30.1%)	71 (13.4%)	
3.8–5.1	487 (77.2%)	68 (66.0%)	(79.4%)	
>5.1	42 (6.7%)	4 (3.9%)	38 (7.2%)	
HGB, (g/L)				<0.001
<115	93 (14.7%)	29 (28.2%)	64 (12.1%)	
115–150	439 (69.6%)	68 (66.0%)	371 (70.3%)	
>150	99 (15.7%)	6 (5.8%)	93 (17.6%)	
LYMPH #, (×10^9^/L)				<0.001
<1.1	154 (24.4%)	47 (45.6%)	107 (20.3%)	
1.1–3.2	462 (73.2%)	52 (50.5%)	410 (77.7%)	
>3.2	15 (2.4%)	4 (3.9%)	11 (2.1%)	
LYMPH, (%)				0.016
<20	194 (30.7%)	44 (42.7%)	150 (28.4%)	
20–50	429 (68.0%)	58 (56.3%)	371 (70.3%)	
>50	8 (1.3%)	1 (1.0%)	7 (1.3%)	
PLT, (×10^9^/L)				0.013
<125	24 (3.8%)	7 (6.8%)	17 (3.2%)	
125–350	547 (86.7%)	93 (90.3%)	454 (86.0%)	
>350	60 (9.5%)	3 (2.9%)	57 (10.8%)	
RBP, (mg/L)				0.026
<25	116 (18.4%)	9 (8.7%)	107 (20.3%)	
25–70	502 (79.6%)	90 (87.4%)	412 (78.0%)	
>70	8 (1.3%)	1 (1.0%)	7 (1.3%)	
CEA, (ng/mL)				0.042
0–10	527 (83.5%)	77 (74.8%)	450 (85.2%)	
>10	86 (13.6%)	20 (19.4%)	66 (12.5%)	
CA125, (U/mL)				0.319
0–35	384 (60.9%)	50 (48.5%)	334 (63.3%)	
>35	173 (27.4%)	28 (27.2%)	145 (27.5%)	
CYFRA211, (ng/mL)				0.822
0.1–6.0	504 (79.9%)	71 (68.9%)	433 (82.0%)	
>6.0	73 (11.6%)	11 (10.7%)	62 (11.7%)	
NSE, (ng/mL)				0.007
0–16.3	133 (21.1%)	31 (30.1%)	102 (19.3%)	
>16.3	477 (75.6%)	65 (63.1%)	412 (78.0%)	
Gender				0.005
Female	168 (26.6%)	16 (15.5%)	152 (28.8%)	
Male	463 (73.4%)	87 (84.5%)	376 (71.2%)	
Smoking history				0.157
Yes	428 (67.8%)	76 (73.8%)	352 (66.7%)	
No	203 (32.2%)	27 (26.2%)	176 (33.3%)	
T stage				0.010
T1	63 (10.0%)	5 (4.9%)	58 (11.0%)	
T2	189 (30.0%)	26 (25.2%)	163 (30.9%)	
T3	101 (16.0%)	12 (11.7%)	89 (16.9%)	
T4	278 (44.1%)	60 (58.3%)	218 (41.3%)	
N stage				0.003
N0	80 (12.7%)	3 (2.9%)	77 (14.6%)	
N1	30 (4.8%)	2 (1.9%)	28 (5.3%)	
N2	408 (64.7%)	74 (71.8%)	334 (63.3%)	
N3	113 (17.9%)	24 (23.3%)	89 (16.9%)	
ECOG				0.009
<2	288 (45.6%)	35 (34.0%)	253 (47.9%)	
≥2	343 (54.4%)	68 (66.0%)	275 (52.1%)	
Clinical stages				<0.001
Limited	435 (68.9%)	0	435 (82.4%)	
Extensive	196 (31.1%)	103 (100%)	93 (17.6%)	

Abbreviations: #, absolute value; BM, brain metastases; CA125, carbohydrate antigen 125; CEA, carcinoembryonic antigen; CYFRA211, non‐small cell lung cancer associated antigen; ECOG, Eastern Cooperative Oncology Group; HGB, haemoglobin; LYMPH, lymphocyte; N, node; NSE, neuron‐specific enolase; PLT, platelet; RBC, red blood cell; RBP, retinol‐binding protein; SCLC, small cell lung cancer; T, tumour; WBC, white blood cell.

### 3.2 Identification and selection of predictors

**FIGURE 2:**
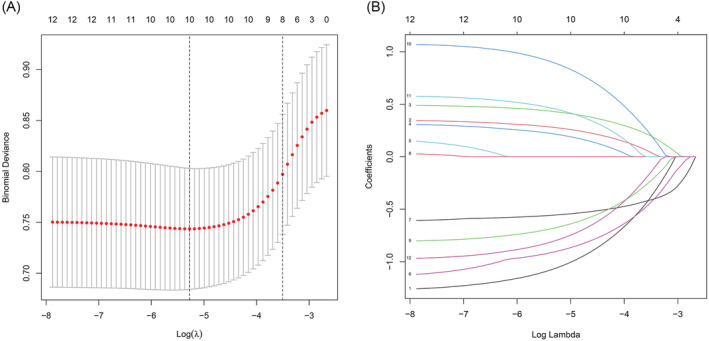
Predictive characteristics screening by the least absolute shrinkage and selection operator (LASSO) logistic regression model. (A) Appropriate parameter (λ) selection in the LASSO applied 10‐fold cross‐validation via minimum criteria. The partial likelihood deviance (binomial deviance) curve was plotted versus log (λ). And dotted vertical lines were drawn at the optimal values applying the minimum criteria and the 1 standard error (SE) of the minimum criteria (the 1‐SE criteria). (B) LASSO coefficient profiles of the 10 variables, which were selected as potential predictors of SCLC patients with brain metastases (BM).

Univariate logistic regression analysis identified significant indices, including gender, T stage, N stage, ECOG, RBC, HGB, LYMPH #, PLT, RBP, CEA, NSE, PLR, and NLR, and the percentages of lymphocytes (*p* < 0.05) (Table S2). After we took missing values (removed by R language before the analysis), actual clinical significance and confounding factors into consideration, the risk factors including gender, T stage, N stage, ECOG, RBC, HGB, LYMPH #, PLT, RBP, CEA, NSE and the percentages of lymphocytes were finally selected for subsequent LASSO regression analysis. Finally, we obtained ten features with non‐zero coefficients as potential predictors by the LASSO analysis (Figure 2A,B). These predictors included gender (OR = 0.284, 95% CI: 0.135–0.553, *p* < 0.001), HGB (OR = 0.356, 95% CI: 0.213–0.583, *p* < 0.001), NSE (OR = 0.378, 95% CI: 0.211–0.680, *p* = 0.001), PLT (OR = 0.447, 95% CI: 0.214–0.896, *p* = 0.028), LYMPH # (OR = 0.551, 95% CI: 0.332–0.914, *p* = 0.021), ECOG (OR = 1.347, 95% CI: 0.815–2.250, *p* = 0.249), T stage (OR = 1.407, 95% CI: 1.093–1.830, *p* = 0.009), N stage (OR = 1.638, 95% CI: 1.174–2.358, *p* = 0.005), CEA (OR = 1.788, 95% CI: 0.918–3.381, *p* = 0.079) and RBP (OR = 2.977, 95% CI: 1.505–6.315, *p* = 0.003) (Table S3).

### 3.3 Construction of a nomogram for predicting the probability of BM in SCLC patients

**FIGURE 3:**
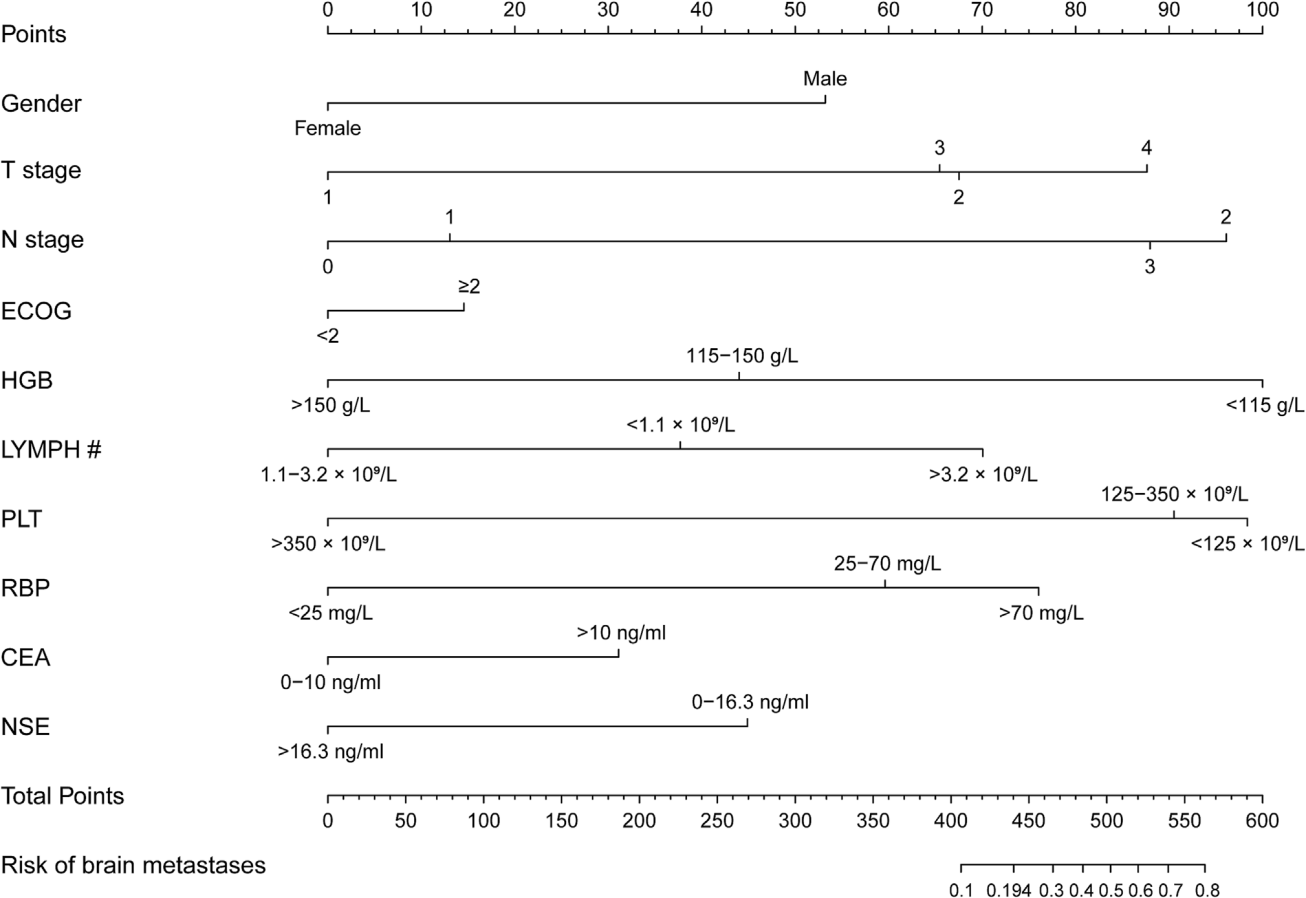
Nomogram for predicting brain metastases (BM) in small cell lung cancer (SCLC) patients. In the use of nomogram, we shall draw a vertical line to the reference line to determine the score of each predictive value, sum the respective scores, and then draw a vertical line from the total point line to figure out the predictive probability of BM.

The above 10 predictive factors were used to construct a visualized nomogram (Figure 3). The predicted risk points for each variable in the nomogram are displayed in Table S4. Clinicians could easily calculate a total score for an individual SCLC patient by summing each single item score located in the total point axis, which is further converted to the probability of BM occurrence by drawing a vertical line across the total score (see the bottom scale in Figure 3). Specifically, the prediction of BM risk in SCLC patients using the nomogram model is performed as follows: (1) determine the individual score of each predictor on the scale; (2) calculate the total score of 10 predictors; and (3) draw a vertical line from the total score line to find out the risk of BM.

A SCLC individual with a risk score >0.194 was regarded as a high‐risk patient for BM. For example, if a SCLC patient that was a male, combined with T2N2, ECOG ≥2, HGB <115 g/L, LYMPH # <1.1 × 10^9^/L, PLT <125 × 10^9^/L, RBP >70 mg/L, CEA >10 ng/mL and NSE >16.3 ng/mL, was defined to be 0.839 (95% CI: 0.250–0.988), which was higher than the risk score, and the patients were classified to the high‐risk group. In addition, a SCLC male with T1N2, ECOG <2, HGB <115g/L, LYMPH # between 1.1 and 3.2 × 10^9^/L, PLT between 125 and 350 × 10^9^/L, RBP <25 mg/L, CEA between 0 and 10 ng/mL and NSE >16.3 ng/mL was defined to be 0.024 (95% CI: 0.005–0.105), which was lower than 0.194, the patients were classified to the low‐risk group. As identifying higher than the cut‐off value, this SCLC patient was the high‐risk group that could provide more direct information for clinicians to take early intervention.

### 3.4 Performance and validation of predictive model

**FIGURE 4:**
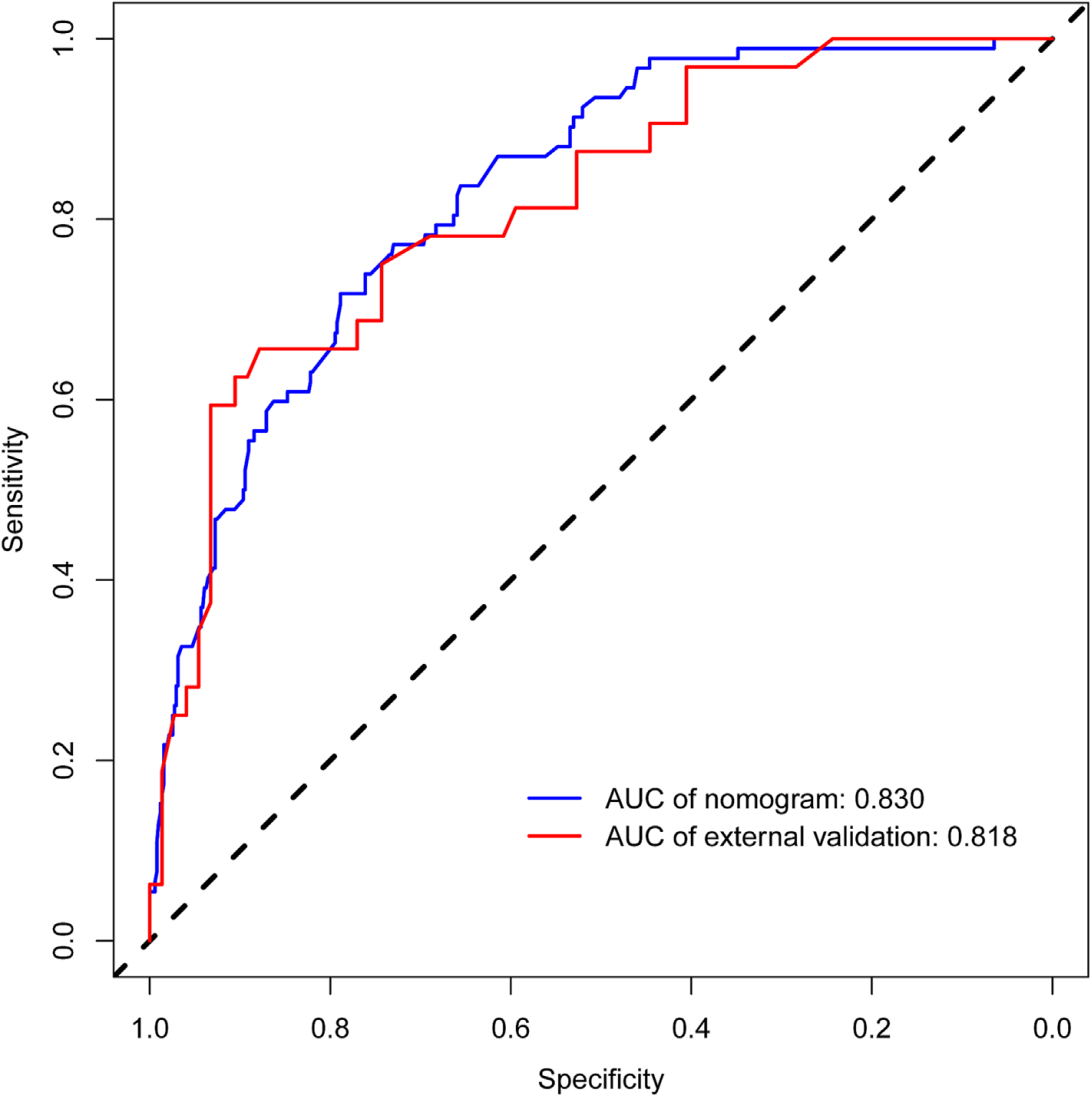
The receiver operating characteristic (ROC) curve analysis to predict brain metastases (BM) in small cell lung cancer (SCLC) patients. AUC, area under the ROC curve.

**FIGURE 5:**
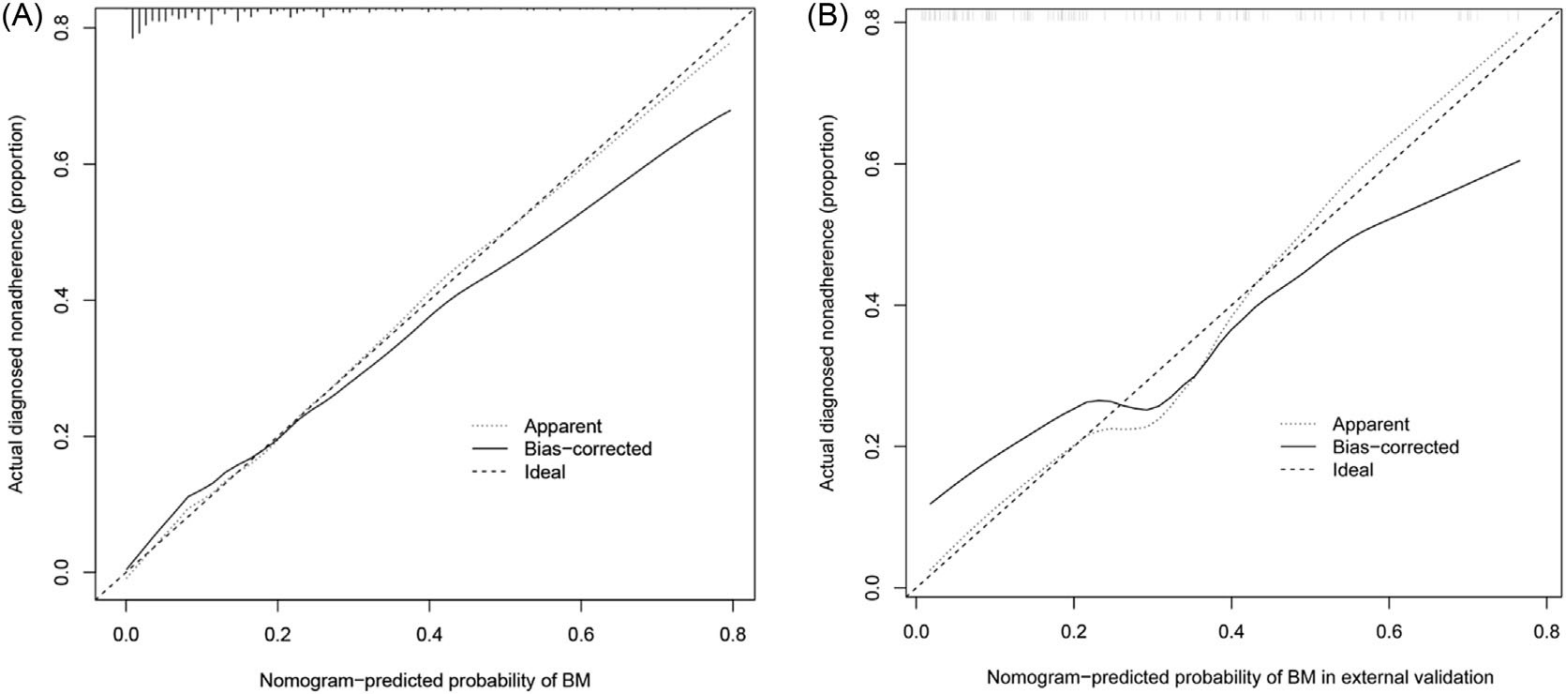
Calibration curves of the nomogram for predicting brain metastases (BM) in small cell lung cancer (SCLC) patients. The x‐axis and y‐axis represent the predicted BM and the actually diagnosed BM, respectively. The diagonal dotted line represents a perfect prediction of an ideal model. The solid line represents the performance of the nomogram, where closer proximity to the diagonal dashed line indicates a better prediction. (A) The training cohort, (B) the external validation cohort.

**FIGURE 6:**
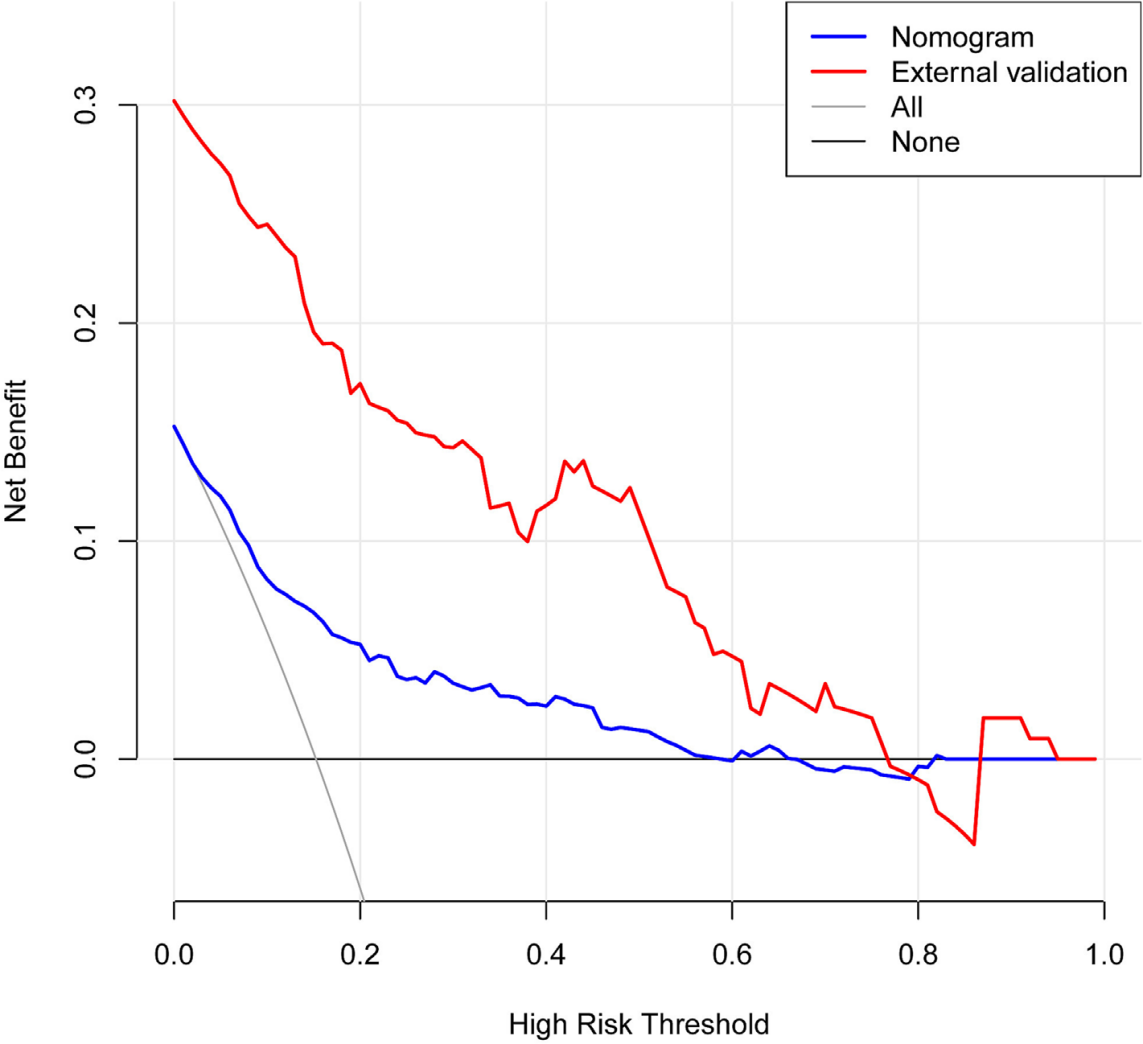
Decision curve analysis (DCA) for the nomogram. The y‐axis means the net benefit, whereas the blue line represents the nomogram model. The grey and black lines display the assumption that all patients and no patients have brain metastases (BM), respectively.

The C‐index of this predictive model was 0.830 and the AUC was also 0.830 (95% CI: 0.788–0.872), indicating that the model possessed a good discriminative ability. Moreover, the optimal cut‐off value of the nomogram was 0.194 according to the Youden's method. The specificity and sensitivity of this model were 78.9% and 71.7%, respectively (Figure 4). In addition, the C‐indices were 0.788 and 0.818 in internal validation by 1000 bootstrap resamples and external validation, respectively, which also showed a good discriminative performance. The calibration plot of the nomogram model is presented in Figure 5, which reveals an excellent agreement between the observed outcome frequencies and the predicted probabilities of BM. The *p*‐value of 0.562 for the Hosmer–Lemeshow test further indicated ideal results. In addition, the results of DCA showed a better net benefit with a broader range of threshold probability with a net clinical benefit of 1%–58% (Figure 6).

### 3.5 Model presentation

A free web calculator based on the nomogram model was built and is available at https://dynnomapp.shinyapps.io/dynnomapp/.

## 4 DISCUSSION

SCLC is characterized by high proliferation rate and metastatic risk. Therefore, several studies have focused on its mechanisms, independent risk factors and treatment strategies.^15^, ^16^, ^17^, ^18^, ^19^

Compared with patients carrying other common solid tumours, SCLC patients, especially those with BM, exhibit higher mortality. Therefore, prediction of BM is an important part of further management. PCI is an essential method for controlling BM, but it is not recommended in all SCLC patients due to the side effects such as worsening physical status and neurocognitive impairment.^7^, ^8^ Therefore, it is essential to identify the high‐risk population of BM early and implement PCI treatment. However, there is still a lack of effective means for early detection of BM in SCLC patients, especially in the early stage. Previous studies have tried to find reliable predictors for BM, such as CEA and programmed cell death‐Ligand 1 (PD‐L1).^20^, ^21^ Recent studies have found that immune checkpoint inhibitors may prolong progression‐free survival (PFS) and reduce BM risk in patients with SCLC, which also suggested that immediate identification of high‐risk individuals for BM in the future may benefit these people by adding immune checkpoint inhibitors in advance, and it is essential to predict the occurrence of BM.^20^ Although the above biomarkers have certain predictive ability for BM, there is still a lack of accurate and systematic decision‐making methods in clinical application to identify high‐risk SCLC individuals for BM. Our study comprehensively incorporated clinically common indices to develop and validate a predictive model to predict BM in SCLC patients.

In this study, we constructed a nomogram to predict the probability of BM in SCLC patients. We eventually identified 10 factors, including gender, T stage, N stage, ECOG, HGB, LYMPH #, PLT, RBP, CEA and NSE, which are readily available in clinical practice. According to our nomogram, the probability of BM exceeded 70% if the score was 540 or higher in patients with SCLC. Some of the included indicators in our model are consistent with the previous findings. A few studies demonstrated that a gender of male could predict increased BM risk and a gender of female was significantly associated with longer BM‐free and overall survival (OS), as well as with a lower incidence of metachronous brain failure.^22^ Zeng et al. demonstrated stage IIIB‐IV (TNM classification system 8th edition) as an independent risk factor to be significantly associated with BM after PCI in SCLC.^23^ Previous study also showed that high serum CEA value was an independent prognostic factor for BM development in SCLC patients.^21^ Guo Dong et al. demonstrated that due to the ability to penetrate the blood–brain barrier and adhesion between vascular tumour cells, high CEA expression could promote BM development.^21^ NSE, a glycolytic enzyme, secreted from nerve and neuroendocrine cells, is currently the most commonly used biomarker for SCLC. Furthermore, a study revealed that elevated serum NSE at relapse in SCLC patients with BM was lower than that in patients without BM, it may support our result. In addition, the clinical performance status assessed according to the ECOG score is a significant prognostic factor for SCLC. A previous study has shown that ECOG is one of the most powerful prognostic factors and it could independently affect the OS of SCLC patients with BM.^24^ Indeed, a retrospective analysis revealed that ECOG was also an important risk factor for BM in SCLC patients.^25^ These results are in line with our findings.

Although the above studies revealed independent risk factors for BM in SCLC patients, no study has conducted a systematic model construction. Other studies analysed risk factors for BM, and the results were not exactly the same as our study. We supposed that the different inclusion and exclusion criteria adopted by the studies contributed to the heterogeneity. Even after the previous multivariate analysis, a shift towards visualization and assisting clinical decision‐making has not been achieved. This study innovatively used a clinical prediction model to perform LASSO regression analysis, which was used to minimize the risk of overfitting and contributed to the development of the optimal model. Furthermore, a visualized nomogram and a free‐accessed web calculator were constructed. We found some indicators innovatively to participate the development of BM in SCLC patients: HGB, PLT, LYMPH # and RBP. Low HGB levels and PLT counts were identified as an adverse prognostic factor in BM from solid extracranial cancers.^26^ Low HGB leads to tumour hypoxia,^27^ and sustained tumour hypoxia could increase proclivity for distant metastasis.^28^ Platelet inhibitor clopidogrel use as an anti‐cancer drug reported clopidogrel treatment increased the risk of metastasis in mice, but the mechanism behind this effect remains to be clarified.^29^ The decrease or increase of LYMPH # caused by lymphocyte dysfunction increased the risk of BM in varying degrees; however, its specific role and mechanism in BM remain unclear. RBP is widely circulated in blood, urine, cerebrospinal fluid and other body fluids, and its rise can be observed in tumour patients. The RBP family, especially RBP4, has been implicated to be associated with tumour invasion and metastases, which could involve the hypermethylation in the gene body.^30^ Therefore, these were consistent with our results that the higher RBP levels predict the higher risk BM.

The prediction model was validated to have good performance in the clinic. The C‐index was 0.830 with a specificity of 78.9% and a sensitivity of 71.7%, indicating good discriminative ability. And DCA showed a better net benefit with a wider threshold probability. PCI is considered to be the standard treatment of SCLC and could extend the limited stage small cell lung cancer OS. However, it is not clear in patients with stage I‐II SCLC with low risk of brain metastasis and patients ≥70 years old or in poor health. Therefore, risk assessment should be individualized, and treatment decisions should be discussed with patients. This predictive model could help doctors predict that SCLC patients have high risk for BM and correspondingly develop appropriate therapeutic strategies.

This study has the following limitations. First, this study was a retrospective study and cannot guarantee the integrity of the data, resulting in a lack of some valuable indicators, such as metastatic sites, pro‐gastrin‐releasing peptide (ProGRP) and treatment (chemotherapy, immune checkpoint inhibitors [ICIs] or radiotherapy). Second, our study had no further survival data to investigate about the differential prognosis between high‐ and low‐risk of BM subgroups. Therefore, rigorous and prospective cohorts with larger sample size are needed. Meanwhile, basic experiments should also be used to explore the key steps in SCLC BM.

## 5 CONCLUSIONS

In conclusion, we constructed a visualized nomogram to predict the risk of BM in patients with SCLC, which covered common clinical indicators and showed good discriminative performance. We believe that the nomogram established in this study will assist clinicians in clinical decision‐making regarding BM and ultimately provide more benefit to the high‐risk population.

## AUTHOR CONTRIBUTIONS

**Weiwei Li**: Conceptualization; investigation; writing—original draft; writing—review and editing; data collection and interpretation. **Can Ding**: Conceptualization; methodology; resources; and statistical analysis. **Wei Sheng**, **Qiang Wan**, **Zhengguo Cui** and **Guiye Qi**: Provide expert advice and project supervision. **Yi Liu**: Supervised the study planning and design; and statistical analysis.

## CONFLICT OF INTEREST STATEMENT

The authors declare no conflicts of interest.

## ETHICS STATEMENT

The studies involving human participants were reviewed and approved by the Shandong Provincial Hospital Medical Ethics Committee (Ethical Review of Medical Research on Human Being No. 2020‐301). Written informed consent for participation was not required for this study in accordance with the national legislation and the institutional requirements.

## Supporting information


**Table S1.** Baseline features of SCLC patients with BM and without BM subgroups.
**Table S2.** Univariate logistic regression analysis for brain metastases in patients with SCLC.
**Table S3.** Identification of predicted variables for nomogram.
**Table S4.** Predictive risk points of each variable in the nomogram.

**Data S1.** TRIPOD Checklist. Prediction Model Development and Validation

## Data Availability

The raw data supporting the conclusions of this article will be made available by the authors, without undue reservation.
