# Supplementary material for: Development and validation of a nomogram for the prediction of brain metastases in small cell lung cancer
Source: Clin Respir J. 2023 Apr 18;17(5):456–67. doi: 10.1111/crj.13615 (PMC10214575; doi:10.1111/crj.13615)
Supplement: Supplementary file 1 — Table S1. Baseline features of SCLC patients with BM and without BM subgroups. Table S2. Univariate logistic regression analysis for brain metastases in patients with SCLC. Table S3. Identification of predicted variables for nomogram. Table S4. Predictive risk points of each variable in the nomogram. [file CRJ-17-456-s001.docx]

Supplementary Material

# Supplementary Tables

**Table S1.** Baseline features of SCLC patients with BM and without BM subgroups.

| **Parameters** | **Total**  **(N = 631)** | **With BM**  **(N = 103)** | **Without BM**  **(N = 528)** | ***P-*value** |
| --- | --- | --- | --- | --- |
| **Age, (years)** |  |  |  | 0.239 |
| **median [IQR]** | 61 [54-66] | 60 [53-65] | 61 [54-67] |  |
| **WBC, (× 10^9^/L)** |  | | | 0.210 |
| <3.5 | 42 (6.7%) | 10 (9.7%) | 32 (6.1%) |  |
| 3.5–9.5 | 532 (84.3%) | 87 (84.5%) | 445 (84.3%) |  |
| >9.5 | 57 (9.0%) | 6 (5.8%) | 51 (9.7%) |  |
| **RBC, (× 10^12^/L)** |  | | | < 0.001 |
| <3.8 | 102 (16.2%) | 31 (30.1%) | 71 (13.4%) |  |
| 3.8–5.1 | 487 (77.2%) | 68 (66.0%) | 1. (79.4%) |  |
| >5.1 | 42 (6.7%) | 4 (3.9%) | 38 (7.2%) |  |
| **HGB, (g/L)** |  | | | < 0.001 |
| <115 | 93 (14.7%) | 29 (28.2%) | 64 (12.1%) |  |
| 115–150 | 439 (69.6%) | 68 (66.0%) | 371 (70.3%) |  |
| >150 | 99 (15.7%) | 6 (5.8%) | 93 (17.6%) |  |
| **NEUT #, (× 10^9^/L)** |  | | | 0.435 |
| <1.8 | 38 (6.0%) | 7 (6.8%) | 31 (5.9%) |  |
| 1.8–6.3 | 514 (81.5%) | 87 (84.5%) | 427 (80.9%) |  |
| >6.3 | 79 (12.5%) | 9 (8.7%) | 70 (13.3%) |  |
| **NEUT, (%)** |  | | | 0.175 |
| <40 | 10 (1.6%) | 0 (0%) | 10 (1.9%) |  |
| 40–75 | 525 (83.2%) | 83 (80.6%) | 442 (83.7%) |  |
| >75 | 96 (15.2%) | 20 (19.4%) | 76 (14.4%) |  |
| **LYMPH #, (× 10^9^/L)** |  | | | < 0.001 |
| <1.1 | 154 (24.4%) | 47 (45.6%) | 107 (20.3%) |  |
| 1.1–3.2 | 462 (73.2%) | 52 (50.5%) | 410 (77.7%) |  |
| >3.2 | 15 (2.4%) | 4 (3.9%) | 11 (2.1%) |  |
| **LYMPH, (%)** |  | | | 0.016 |
| <20 | 194 (30.7%) | 44 (42.7%) | 150 (28.4%） |  |
| 20–50 | 429 (68.0%) | 58 (56.3%) | 371 (70.3%) |  |
| >50 | 8 (1.3%) | 1 (1.0%) | 7 (1.3%) |  |
| **PLT, (× 10^9^/L)** |  | | | 0.013 |
| <125 | 24 (3.8%) | 7 (6.8%) | 17 (3.2%) |  |
| 125–350 | 547 (86.7%) | 93 (90.3%) | 454 (86.0%) |  |
| >350 | 60 (9.5%) | 3 (2.9%) | 57 (10.8%) |  |
| **MONO #, (× 10^9^/L)** |  | | | 0.283 |
| <0.1 | 8 (1.3%) | 2 (1.9%) | 6 (1.1%) |  |
| 0.1–0.6 | 444 (70.4%) | 78 (75.7%) | 366 (69.3%) |  |
| >0.6 | 179 (28.4%) | 23 (22.3%) | 156 (29.5%) |  |
| **MONO, (%)** |  | | | 0.776 |
| <3 | 25 (4.0%) | 3 (2.9%) | 22 (4.2%) |  |
| 3–10 | 476 (75.4%) | 80 (77.7%) | 396 (75.0%) |  |
| >10 | 130 (20.6%) | 20 (19.4%) | 110 (20.8%) |  |
| **D–dimer, (mg/L)** |  | | | 0.092 |
| 0–0.5 | 321 (50.9%) | 43 (41.7%) | 278 (52.7%) |  |
| >0.5 | 300 (47.5%) | 55 (53.4%) | 245 (46.4%) |  |
| **AST, (U/L)** |  | | | 0.838 |
| <13 | 13 (2.1%) | 2 (1.9%) | 11 (2.1%) |  |
| 13–35 | 553 (87.6%) | 90 (87.4%) | 463 (87.7%) |  |
| >35 | 60 (9.5%) | 8 (7.8%) | 52 (9.8%) |  |
| **ALT, (U/L)** |  | | | 0.595 |
| <7 | 10 (1.6%) | 1 (1.0%) | 9 (1.7%) |  |
| 7–40 | 551 (87.3%) | 91 (88.3%) | 460 (87.1%) |  |
| >40 | 65 (10.3%) | 8 (7.8%) | 57 (10.8%) |  |
| **ALP, (U/L)** |  | | | 0.725 |
| ≤140 | 590 (93.5%) | 95 (92.2%) | 495 (93.8%) |  |
| >140 | 36 (5.7%) | 5 (4.9%) | 31 (5.9%) |  |
| **ADA, (U/L)** |  | | | 0.451 |
| 4–24 | 622 (98.6%) | 99 (96.1%) | 523 (99.1%) |  |
| >24 | 3 (0.5%) | 0 (0%) | 3 (0.6%) |  |
| **SA, (mg/L)** |  | | | 0.118 |
| <456 | 18 (2.9%) | 1 (1.0%) | 17 (3.2%) |  |
| 456–754 | 468 (74.2%) | 82 (79.6%) | 386 (73.1%) |  |
| >754 | 138 (21.9%) | 16 (15.5%) | 122 (23.1%) |  |
| **GLDH, (U/L)** |  | | | 0.916 |
| 0–7.4 | 540 (85.6%) | 86 (83.5%) | 454 (86.0%) |  |
| >7.4 | 84 (13.3%) | 13 (12.6%) | 71 (13.4%) |  |
| **ALB, (g/L)** |  | | | 0.793 |
| <40 | 314 (49.8%) | 49 (47.6%) | 265 (50.2%) |  |
| 40–55 | 310 (49.1%) | 51 (49.5%) | 259 (49.1%) |  |
| >55 | 2 (0.3%) | 0 (0%) | 2 (0.4%) |  |
| **GLO, (g/L)** |  | | | 0.819 |
| <20 | 12 (1.9%) | 2 (1.9%) | 10 (1.9%) |  |
| 20–40 | 603 (95.6%) | 97 (94.2%) | 506 (95.8%) |  |
| >40 | 11 (1.7%) | 1 (1.0%) | 10 (1.9%) |  |
| **A/G** |  | | | 0.200 |
| <1.2 | 143 (22.7%) | 18 (17.5%) | 125 (23.7%) |  |
| 1.2–2.4 | 481 (76.2%) | 81 (78.6%) | 400 (75.8%) |  |
| >2.4 | 2 (0.3%) | 1 (1.0%) | 1 (0.2%) |  |
| **SOD, (U/ml)** |  | | | 0.356 |
| <129 | 105 (16.6%) | 12 (11.7%) | 93 (17.6%) |  |
| 129–216 | 515 (81.6%) | 86 (83.5%) | 429 (81.3%) |  |
| >216 | 4 (0.6%) | 1 (1.0%) | 3 (0.6%) |  |
| **GLU, (mmol/l)** |  | | | 0.384 |
| <3.9 | 6 (1.0%) | 78 (75.7%) | 6 (1.1%) |  |
| 3.9–6.3 | 499 (79.1%) | 23 (22.3%) | 421 (79.7%) |  |
| >6.3 | 122 (19.3%) | 0 (0%) | 99 (18.8%) |  |
| **CRP, (mg/L)** |  | | | 0.390 |
| 0–10 | 434 (68.8%) | 73 (70.9%) | 361 (68.4%) |  |
| >10 | 185 (29.3%) | 26 (25.2%) | 159 (30.1%) |  |
| **BMG, (mg/L)** |  | | | 0.315 |
| <1.0 | 9 (1.4%) | 0 (0%) | 9 (1.7%) |  |
| 1.0–3.0 | 578 (91.6%) | 92 (89.3%) | 486 (92.0%) |  |
| >3.0 | 39 (6.2%) | 8 (7.8%) | 31 (5.9%) |  |
| **RBP, (mg/L)** |  | | | 0.026 |
| <25 | 116 (18.4%) | 9 (8.7%) | 107 (20.3%) |  |
| 25–70 | 502 (79.6%) | 90 (87.4%) | 412 (78.0%) |  |
| >70 | 8 (1.3%) | 1 (1.0%) | 7 (1.3%) |  |
| **C1q, (mg/L)** |  | | | 0.201 |
| <159 | 122 (19.3%) | 16 (15.5%) | 106 (20.1%) |  |
| 159–233 | 402 (63.7%) | 72 (69.9%) | 330 (62.5%) |  |
| >233 | 102 (16.2%) | 12 (11.7%) | 90 (17.0%) |  |
| **Na^+^, (mmol/L)** |  | | | 0.339 |
| <137 | 162 (25.7%) | 22 (21.4%) | 140 (26.5%) |  |
| ≥137 | 465 (73.7%) | 78 (75.8%) | 387 (73.3%) |  |
| **CO_2_, (mmol/L)** |  | | | 0.493 |
| <20 | 12 (1.9%) | 3 (2.9%) | 9 (1.7%) |  |
| 20–30 | 583 (92.4%) | 93 (90.3%) | 490 (92.8%) |  |
| >30 | 29 (4.6%) | 3 (2.9%) | 26 (4.9%) |  |
| **CEA, (ng/ml)** |  | | | 0.042 |
| 0–10 | 527 (83.5%) | 77 (74.8%) | 450 (85.2%) |  |
| >10 | 86 (13.6%) | 20 (19.4%) | 66 (12.5%) |  |
| **CA125, (U/ml)** |  | | | 0.319 |
| 0–35 | 384 (60.9%) | 50 (48.5%) | 334 (63.3%) |  |
| >35 | 173 (27.4%) | 28 (27.2%) | 145 (27.5%) |  |
| **CYFRA211, (ng/ml)** |  | | | 0.822 |
| 0.1–6.0 | 504 (79.9%) | 71 (68.9%) | 433 (82.0%) |  |
| >6.0 | 73 (11.6%) | 11 (10.7%) | 62 (11.7%) |  |
| **NSE, (ng/ml)** |  | | | 0.007 |
| 0–16.3 | 133 (21.1%) | 31 (30.1%) | 102 (19.3%) |  |
| >16.3 | 477 (75.6%) | 65 (63.1%) | 412 (78.0%) |  |
| **Gender** |  | | | 0.005 |
| Female | 168 (26.6%) | 16(15.5%) | 152 (28.8%) |  |
| Male | 463 (73.4%) | 87 (84.5%) | 376 (71.2%) |  |
| **Smoking history** |  | | | 0.157 |
| Yes | 428 (67.8%) | 76 (73.8%) | 352 (66.7%) |  |
| No | 203 (32.2%) | 27 (26.2%) | 176 (33.3%) |  |
| **Weigh down** |  | | | 0.305 |
| Yes | 121 (19.2%) | 16 (15.5%) | 105 (19.9%) |  |
| No | 510 (80.8%) | 87 (84.5%) | 423 (80.1%) |  |
| **Primary lesion** |  | | | 0.136 |
| Peripheral | 146 (23.1%) | 18 (17.5%) | 128 (24.2%) |  |
| Central | 485 (76.9%) | 85 (82.5%) | 400 (75.8%) |  |
| **T stage** |  | | | 0.010 |
| T1 | 63 (10.0%) | 5 (4.9%) | 58 (11.0%) |  |
| T2 | 189 (30.0%) | 26 (25.2%) | 163 (30.9%) |  |
| T3 | 101 (16.0%) | 12 (11.7%) | 89 (16.9%) |  |
| T4 | 278 (44.1%) | 60 (58.3%) | 218 (41.3%) |  |
| **N stage** |  | | | 0.001 |
| N0 | 80 (12.7%) | 3 (2.9%) | 77 (14.6%) |  |
| N1 | 30 (4.8%) | 2 (1.9%) | 28 (5.3%) |  |
| N2 | 408 (64.7%) | 74 (71.8%) | 334 (63.3%) |  |
| N3 | 113 (17.9%) | 24 (23.3%) | 89 (16.9%) |  |
| **ECOG** |  | | | 0.009 |
| < 2 | 288 (45.6%) | 35 (34.0%) | 253 (47.9%) |  |
| ≥ 2 | 343 (54.4%) | 68 (66.0%) | 275 (52.1%) |  |
| **mGPS** |  | | | 0.518 |
| 0 | 456 (72.3%) | 79 (76.7%) | 377 (71.4%) |  |
| 1 | 115 (18.2%) | 15 (14.6%) | 100 (18.9%) |  |
| 2 | 60 (9.5%) | 9 (8.7%) | 51 (9.7%) |  |
| **PLR, median [IQR]** | 154.84 [113.64-226.71] | 183.43 [111.18-257.14] | 152.91 [114.09-221.75] | 0.156 |
| **NLR, median [IQR]** | 2.56 [1.77-3.81] | 2.91 [1.70-4.65] | 2.52 [1.77-3.69] | 0.065 |
| **LMR, median [IQR]** | 3.29 [2.18-4.66] | 2.92 [1.74-4.42] | 3.41 [2.24-4.70] | 0.072 |
| **SII, median [IQR]** | 590.96 [387.83-909.12] | 628.56 [331.55-905.91] | 589.89 [398.26-910.94] | 0.714 |
| **AAPR, median [IQR]** | 0.43 [0.36-0.53] | 0.44 [0.37-0.55] | 0.43 [0.36-0.52] | 0.354 |

Abbreviations: SCLC, Small Cell Lung Cancer; BM, Brain metastases; WBC, white blood cell; RBC, red blood cell; HGB, hemoglobin; #, absolute value; NEUT, neutrophils; LYMPH, lymphocyte; MONO, monocyte; PLT, platelet; AST, Aspartate aminotransferase; ALT, Alanine aminotransferase; ALP, alkaline phosphatase; ADA, Adenosine deaminase; SA, Sialic acid; GLDH, Glutamate dehydrogenase; ALB, Albumin; GLO, Globulin; A/G, ALB/GLO; SOD, Superoxide dismutase; GLU, glucose; CRP, C-reactive protein; BMG, β2-microglobulin; RBP, Retinol-Binding Protein; C1q, Complement C1q; CO_2_, Nitrogen dioxide; CEA, carcinoembryonic antigen; CA125, carbohydrate antigen 125; CYFRA211, Non-small cell lung cancer associated antigen; NSE, neuron-specific enolase; T, tumor; N, node; ECOG, Eastern Cooperative Oncology Group; mGPS, inflammation-based prognostic scores; PLR, platelet-to-lymphocyte ratio; NLR, neutrophil-to-lymphocyte ratio; LMR, lymphocyte-to-monocyte ratio; SII, systemic immune inflammation index; AAPR, albumin-to-alkaline phosphatase ratio.

**Table S2.** Univariate logistic regression analysis for brain metastases in patients with SCLC.

| **Variables** | **β** | **OR** | **95% CI** | ***P*-value** |
| --- | --- | --- | --- | --- |
| Age, (years) | -0.012 | 0.988 | 0.966-1.009 | 0.263 |
| WBC, (× 10^9^/L) | -0.486 | 0.615 | 0.358-1.058 | 0.079 |
| RBC, (× 10^12^/L) | -0.888 | 0.412 | 0.264-0.641 | < 0.001 |
| HGB, (g/L) | -0.946 | 0.388 | 0.260-0.580 | < 0.001 |
| NEUT#, (× 10^9^/L) | -0.306 | 0.736 | 0.444-1.221 | 0.235 |
| NEUT, (%) | 0.433 | 1.543 | 0.923-2.577 | 0.098 |
| LYMPH#, (× 10^9^/L) | -1.007 | 0.365 | 0.238-0.562 | < 0.001 |
| LYMPH, (%) | -0.604 | 0.546 | 0.358-0.835 | 0.005 |
| PLT, (× 10^9^/L) | -0.965 | 0.381 | 0.200-0.725 | 0.003 |
| MONO#, (× 10^9^/L) | -0.377 | 0.686 | 0.429-1.097 | 0.116 |
| MONO, (%) | -0.007 | 0.993 | 0.631-1.561 | 0.974 |
| D–Dimer, (mg/L) | 0.372 | 1.451 | 0.940-2.241 | 0.093 |
| AST, (U/L) | -0.166 | 0.847 | 0.438-1.637 | 0.622 |
| ALT, (U/L) | -0.196 | 0.822 | 0.424-1.592 | 0.561 |
| ALP, (U/L) | -0.091 | 0.913 | 0.367-2.272 | 0.845 |
| ADA, (U/L) | -19.538 | 0 | NA | 0.999 |
| SA, (mg/L) | -0.233 | 0.792 | 0.491-1.277 | 0.338 |
| GLDH, (U/L) | -0.034 | 0.967 | 0.512-1.823 | 0.916 |
| ALB, (g/L) | 0.039 | 1.04 | 0.682-1.586 | 0.856 |
| GLO, (g/L) | -0.271 | 0.763 | 0.252-2.312 | 0.632 |
| A/G | 0.391 | 1.479 | 0.859-2.546 | 0.158 |
| SOD, (U/ml) | 0.446 | 1.562 | 0.844-2.890 | 0.155 |
| GLU, (mmol/l) | 0.286 | 1.332 | 0.812-2.184 | 0.256 |
| CRP, (mg/L) | -0.212 | 0.809 | 0.498-1.313 | 0.391 |
| BMG, (mg/L) | 0.474 | 1.607 | 0.777-3.322 | 0.201 |
| RBP, (mg/L) | 0.75 | 2.117 | 1.156-3.878 | 0.015 |
| C1q, (mg/L) | -0.027 | 0.974 | 0.681-1.393 | 0.883 |
| Na^+^, (mmol/L) | 0.302 | 1.353 | 0.810-2.258 | 0.248 |
| CO_2_, (mmol/L) | -0.528 | 0.59 | 0.242-1.438 | 0.246 |
| CEA, (ng/ml) | 0.572 | 1.771 | 1.016-3.087 | 0.044 |
| CA125, (U/ml) | 0.255 | 1.29 | 0.781-2.131 | 0.320 |
| CYFRA211, (ng/ml) | 0.079 | 1.082 | 0.543-2.154 | 0.822 |
| NSE, (ng/ml) | -0.656 | 0.519 | 0.321-0.839 | 0.007 |
| Gender | -0.788 | 0.455 | 0.258-0.801 | 0.006 |
| Smoking history | 0.342 | 1.407 | 0.875-2.263 | 0.158 |
| Weigh down | -0.3 | 0.741 | 0.417-1.316 | 0.306 |
| Primary lesion | 0.413 | 1.511 | 0.875-2.609 | 0.138 |
| T stage | 0.328 | 1.388 | 1.120-1.718 | 0.003 |
| N stage | 0.556 | 1.744 | 1.284-2.367 | < 0.001 |
| ECOG | 0.581 | 1.787 | 1.149-2.781 | 0.010 |
| mGPS | -0.155 | 0.857 | 0.608-1.207 | 0.376 |
| PLR | 0.002 | 1.002 | 1.000-1.003 | 0.035 |
| NLR | 0.062 | 1.064 | 1.006-1.125 | 0.030 |
| LMR | -0.038 | 0.962 | 0.888-1.044 | 0.353 |
| SII | <0.001 | 1 | 1 | 0.631 |
| AAPR | 0.462 | 1.587 | 0.561-4.487 | 0.384 |

Abbreviations: β, beta coefficient; OR, odds ratio; CI, confidence interval; NA, not applicable; SCLC, Small Cell Lung Cancer; BM, Brain metastases; WBC, white blood cell; RBC, red blood cell; HGB, hemoglobin; #, absolute value; NEUT, neutrophils; LYMPH, lymphocyte; MONO, monocyte; PLT, platelet; AST, Aspartate aminotransferase; ALT, Alanine aminotransferase; ALP, alkaline phosphatase; ADA, Adenosine deaminase; SA, Sialic acid; GLDH, Glutamate dehydrogenase; ALB, Albumin; GLO, Globulin; A/G, ALB/GLO; SOD, Superoxide dismutase; GLU, glucose; CRP, C-reactive protein; BMG, β2-microglobulin; RBP, Retinol-Binding Protein; C1q, Complement C1q; CO_2_, Nitrogen dioxide; CEA, carcinoembryonic antigen; CA125, carbohydrate antigen 125; CYFRA211, Non-small cell lung cancer associated antigen; NSE, neuron-specific enolase; T, tumor; N, node; ECOG, Eastern Cooperative Oncology Group; mGPS, inflammation-based prognostic scores; PLR, platelet-to-lymphocyte ratio; NLR, neutrophil-to-lymphocyte ratio; LMR, lymphocyte-to-monocyte ratio; SII, systemic immune inflammation index; AAPR, albumin-to-alkaline phosphatase ratio.

**Table S3.** Identification of predicted variables for nomogram.

| **Intercept and variable** | **β** | **95% CI** | **OR** | ***P-*value** |
| --- | --- | --- | --- | --- |
| Intercept | -1.7952 | 0.037-0.711 | 0.166 | 0.017 |
| HGB, (g/L) | -1.0322 | 0.213-0.583 | 0.356 | < 0.001 |
| LYMPH #, (× 10^9^/L) | -0.5966 | 0.332-0.914 | 0.551 | 0.021 |
| PLT, (× 10^9^/L) | -0.8054 | 0.214-0.896 | 0.447 | 0.028 |
| RBP, (mg/L) | 1.0909 | 1.505-6.315 | 2.977 | 0.003 |
| CEA, (ng/ml) | 0.5811 | 0.918-3.381 | 1.788 | 0.079 |
| NSE, (ng/ml) | -0.9721 | 0.211-0.680 | 0.378 | 0.001 |
| Gender | -1.26 | 0.135-0.553 | 0.284 | < 0.001 |
| T stage | 0.3411 | 1.093-1.830 | 1.407 | 0.009 |
| N stage | 0.4933 | 1.174-2.358 | 1.638 | 0.005 |
| ECOG | 0.2979 | 0.815-2.250 | 1.347 | 0.249 |

Abbreviations: β, beta coefficient; OR, odds ration; CI, confidence interval; HGB, hemoglobin; #, absolute value; LYMPH, lymphocyte; PLT, platelet; RBP, Retinol-Binding Protein; CEA, carcinoembryonic antigen; NSE, neuron-specific enolase; T, tumor; N, node; ECOG, Eastern Cooperative Oncology Group.

**Table S4.** Predictive risk points of each variable in the nomogram.

| **Predict factors** | **Points** |
| --- | --- |
| **Gender** | |
| Male | 53 |
| Female | 0 |
| **T** | |
| T1 | 0 |
| T2 | 68 |
| T3 | 65 |
| T4 | 88 |
| **N** | |
| N0 | 0 |
| N1 | 13 |
| N2 | 96 |
| N3 | 88 |
| **ECOG** | |
| <2 | 0 |
| ≥2 | 15 |
| **HGB** | |
| <115 g/L | 100 |
| 115–150 g/L | 44 |
| >150 g/L | 0 |
| **LYMPH #** | |
| <1.1 × 10^9^/L | 38 |
| 1.1–3.2 × 10^9^/L | 0 |
| >3.2 × 10^9^/L | 70 |
| **PLT** | |
| <125 × 10^9^/L | 98 |
| 125–350 × 10^9^/L | 91 |
| >350 × 10^9^/L | 0 |
| **RBP** | |
| <25 mg/L | 0 |
| 25–70 mg/L | 60 |
| >70 mg/L | 76 |
| **CEA** | |
| 0–10 ng/ml | 0 |
| >10 ng/ml | 31 |
| **NSE** | |
| 0–16.3 ng/ml | 45 |
| >16.3 ng/ml | 0 |

Abbreviations: T, tumor; N, node; ECOG, Eastern Cooperative Oncology Group;

HGB, hemoglobin; #, absolute value; LYMPH, lymphocyte; PLT, platelet; RBP,

Retinol-Binding Protein; CEA, carcinoembryonic antigen; NSE, neuron-specific

enolase.
